# Antioxidant Activity of **β**-Glucan

**DOI:** 10.5402/2012/125864

**Published:** 2012-02-19

**Authors:** Kyoko Kofuji, Ayumi Aoki, Kazufumi Tsubaki, Masanori Konishi, Takashi Isobe, Yoshifumi Murata

**Affiliations:** ^1^Faculty of Pharmaceutical Sciences, Hokuriku University, Ho 3, Kanagawa-Machi, Kanazawa 920-1181, Japan; ^2^New Business Promotion Department, ADEKA Corporation, 7-2-35, Higashiogu, Arakawaku, Tokyo 116-8554, Japan

## Abstract

**β**-Glucans extracted from barley, which mainly contains **β**-(1,3-1,4)-D-glucan, are used extensively as supplements and food additives due to their wide biologic activities, including a reduction in blood lipid level. In this study, the antioxidant activity of **β**-glucan was examined to assess potential new benefits associated with **β**-glucan, because oxidative stress is considered one of the primary causal factors for various diseases and aging. **β**-Glucan extracted from barley was found to possess significant antioxidant activity. The amount of antioxidant activity was influenced by different physiologic properties (e.g., structure and molecular size) of **β**-glucan, which varied depending on the source and extraction method used. The antioxidant activity of **β**-glucan was significantly higher than that of various polymers that are used as food additives. These results indicate that **β**-glucan has promise as a polymeric excipient for supplement and food additive with antioxidant and other benefits, which may contribute to enhancing health and beauty.

## 1. Introduction

*β*-Glucan is a polysaccharide comprised of *β*-linked d-glucose molecules. Various *β*-glucans have been extracted from various sources such as fungi, baker's yeast, barley, oats, and seaweed. The physicochemical properties of *β*-glucans differ depending on characteristics of their primary structure, including linkage type, degree of branching, molecular weight, and conformation (e.g., triple helix, single helix, and random coil structures) [1, 2]. *β*-Glucans extracted from barley, which mainly contains *β*-(1,3-1,4)-d-glucan, have been demonstrated to reduce blood lipid levels, including cholesterol and triglyceride levels [3–5]. The mechanisms by which *β*-glucans reduce blood lipid levels have been shown to include prevention of cholesterol reabsorption by adsorption, elimination of bile acid by adsorption, an increase in bile acid synthesis, and suppression of hepatic cholesterol biosynthesis by short-chain fatty acids produced by fermentation with intestinal bacteria [6–8]. Claims that barley products reduce the danger of coronary heart disease have been endorsed by the Food and Drug Administration of the United States [9]. In addition, *β*-glucans extracted from barley have also been reported to possess various other biologic activities, for example, reducing blood glucose level, enhancing insulin response [10], protecting against stress ulcers [11], and restraining allergic reactions [12]. Furthermore, *β*-glucans extracted from barley have been used in health products as a diet food, because glucan is a dietary fiber. Thus, *β*-glucans extracted from barley have been used extensively as supplements and food additives.

Whole grain products are recommended for healthy diets, as they are recognized sources of dietary fiber. Furthermore, Ragaee et al. [13] demonstrated the antioxidant activity of various cereals such as barley, millet, rye, and sorghum. Oxidative stress is considered one of the primary causal factors for aging and various diseases such as arteriosclerosis, cardiovascular disease, cerebral diseases, diabetes, inflammatory diseases, and cancer [14, 15]. It is thought that scavenging of reactive oxygen species (ROS) is important for the prevention of these diseases. If it were to be shown that *β*-glucan, a natural component of grain, exerts antioxidant activity, the utility of *β*-glucan as a polymeric excipient for supplement or food additive would increase further. However, the antioxidant activity of *β*-glucan has not been extensively investigated.

Therefore, the aim of the present study was to examine the antioxidant activity of *β*-glucan to assess potential new health benefits associated with *β*-glucan as a polymeric excipient for supplement and food additive. The effects of the extraction source, extraction method, and molecular size of *β*-glucan on antioxidant activity were also investigated. Furthermore, the antioxidant activity of *β*-glucan was compared with that of polymers that are commonly used as food additives.

## 2. Materials and Methods

### 2.1. Materials

**Figure 1:**
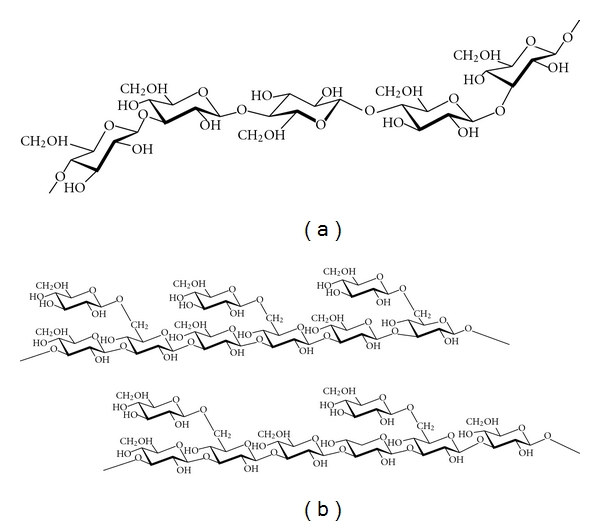
Chemical structure of *β*-glucan: (a) *β*-(1,3-1,4)-d-glucan; (b) *β*-(1,3-1,6)-d-glucan.

*β*-Glucans extracted from barley using different extraction methods and of different molecular sizes, contained more than 70%  *β*-(1,3-1,4)-d-glucan (Figure 1(a)) [16], and *β*-glucan isolated from black yeast (*Aureobasidium pullulans*), which contained more than 85%   *β*-(1,3-1,6)- d-glucan (Figure 1(b)) [1], were donated by ADEKA Co. (Tokyo, Japan). *β*-Glucan extracted from oats, with an unknown composition, was purchased from Megazyme International Ireland Ltd. (Bray, Ireland). Chitosan (Flonac LV) was donated by Kyowa Tecnos Co. Ltd. (Tiba, Japan). Pullulan was donated by Hayashibara Shoji Inc. (Okayama, Japan). Gelatin, pectin from apple, pectin from citrus, curdlan, gellan gum, and xanthan gum were purchased from Wako Pure Chemical Industrial Ltd. (Osaka, Japan). Dextrin and sodium alginate were purchased from Nacalai Tesque Inc. (Kyoto, Japan). All other chemicals were of reagent grade.

### 2.2. Determination of Antioxidant Activity

Concentrations of 0.5–2.0 w/v% of *β*-glucans or other polymers were prepared by dissolving the molecules in ion-exchanged water. Chitosan was dissolved in 0.1 M acetate buffer solution (pH 4.5), because chitosan did not dissolve in ion-exchanged water. Antioxidant activity against hydroxyl radicals in the solution was determined using a Radical Catch kit (ALOKA Co. Ltd., Tokyo, Japan). The kit measures the amount of hydroxyl radicals generated by the Fenton reaction with hydrogen peroxide catalyzed by cobalt using luminol luminescence. Briefly, 50 *μ*L of cobalt chloride reagent, 50 *μ*L of luminal reagent, and 20 *μ*L of sample solution were mixed, and, after preincubation at 37°C for 5 min, 50 *μ*L of hydrogen peroxide reagent was added. The amount of luminescence generated from 80 seconds to 120 seconds after the addition of hydrogen peroxide reagent was detected with a luminescence reader (AccuFLEX Lumi 400; ALOLA Co. Ltd., Tokyo, Japan). Hydroxyl radical scavenging activity was estimated using the following equation:

(1) $$\begin{array}{cc} & \text{Radical}\;\text{scavenge}\;\;\left(\text{\%}\right)\\ & \;\;=\frac{\text{amount}\;\text{of}\;\text{hydroxy}\;\text{radical}\;\text{scavenged}}{\text{amount}\;\text{of}\;\text{hydroxy}\;\text{radical}\;\text{generated}}\times 100. \end{array}$$

## 3. Results and Discussion

### 3.1. Effect of *β*-Glucan Source on Antioxidant Activity

**Figure 2:**
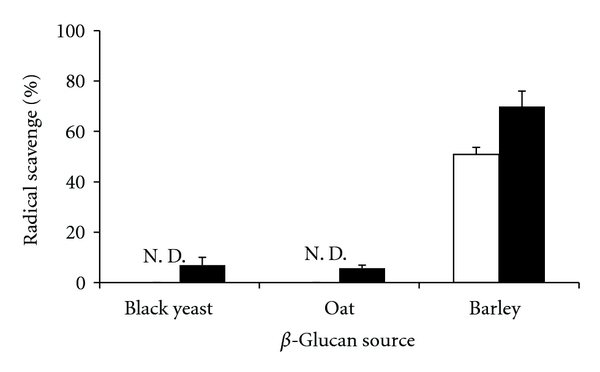
Effect of *β*-glucan source on antioxidant activity. *β*-glucan concentration. □: 0.5 w/v%; ■: 1.0 w/v%. N. D.: not detected. Data are presented as mean ± SD (*n* = 3).

**Figure 3:**
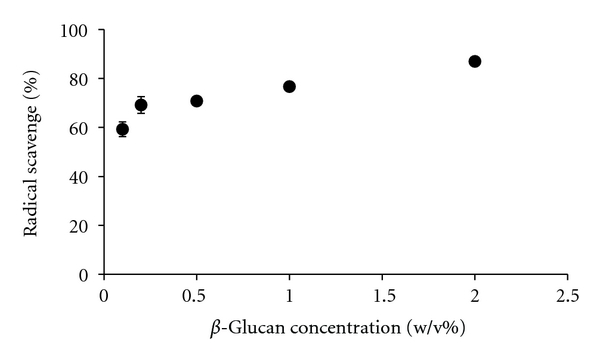
Effect of barley *β*-glucan concentration on antioxidant activity. Data are presented as mean ± SD (*n* = 3).

As shown in Figure 2, the hydroxyl radical scavenging activity of *β*-glucan differed between different sources of *β*-glucan. In particular, *β*-glucan extracted from barley showed the strongest hydroxyl radical scavenging activity. On the other hand, the hydroxyl radical scavenging activity of *β*-glucans extracted from black yeast or oats was fairly low. Furthermore, 0.1 w/v% of *β*-glucan extracted from barley scavenged approximately 60% of the hydroxyl radicals in the system, and the hydroxyl radical scavenging activity of the *β*-glucan increased gradually with an increase in *β*-glucan concentration (Figure 3). *β*-Glucans extracted from both barley or oats were found to comprise mainly *β*-(1,3-1,4)-d-glucan (Figure 1(a)). On the other hand, *β*-glucan extracted from black yeast comprised mainly *β*-(1,3-1,6)-d-glucan (Figure 1(b)). In addition to these differences in linkage type, the properties of *β*-glucans are influenced by the degree of branching, molecular weight, and conformation [1, 2]. Although the mechanisms by which *β*-glucan scavenges hydroxyl radicals are not yet clarified, the different structures of *β*-glucan, which may be associated with the source and extraction method of obtaining *β*-glucan, may influence antioxidant activity.

### 3.2. Effect of Extraction Method for Barley *β*-Glucan on Antioxidant Activity

**Figure 4:**
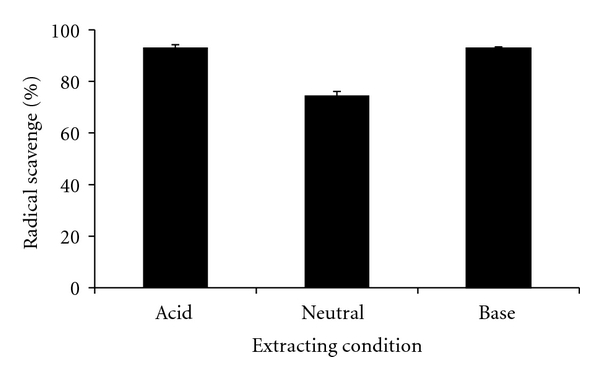
Effect of barley *β*-glucan extraction method on antioxidant activity. *β*-glucan concentration: 1.0 w/v%. Data are presented as mean ± SD (*n* = 3).

*β*-Glucan was extracted from barley using the following basic procedure. First, the barley was crushed and then extracted under neutral, acidic (50 mM citrate buffer aqueous solution (pH 4.0)), or alkaline (50 mM carbonate buffer aqueous solution (pH 9.0)) conditions with warm water at 50°C. After solid-liquid separation, the liquid phase was condensed. The extract was dried, and *β*-glucan was obtained as a powder. The hydroxyl radical scavenging activity of *β*-glucan extracted from barley using the different extraction methods is shown in Figure 4. The hydroxyl radical scavenging activity of *β*-glucans extracted under an acid condition or an alkali condition was slightly higher compared with that when *β*-glucans were extracted under a neutral condition with warm water. The *β*-glucans extracted from barley used in this study comprised more than 70%  *β*-(1,3-1,4)-d-glucan, but also contained other components (i.e., proteins, lipids, saccharides, and dietary fiber). The hydroxyl radical scavenging activity of almost pure *β*-(1,3-1,4)-d-glucan, which was obtained by repeated recrystallization of *β*-glucan extracted under neutral conditions with warm water (50°C), was 43 ± 1%. Although the possibility that other components contributed to the radical scavenging activity of *β*-glucan cannot be completely ruled out, this finding shows that the greater part of the hydroxyl radical scavenging activity was caused by *β*-(1,3-1,4)-d-glucan. However, *β*-glucan extract is generally used without further refinement for supplements or food additives. These findings demonstrate that *β*-glucans extracted using a variety of extraction methods, from acidic to alkaline conditions, have high radical scavenging activity.

### 3.3. Effect of *β*-Glucan Molecular Size on Antioxidant Activity

**Figure 5:**
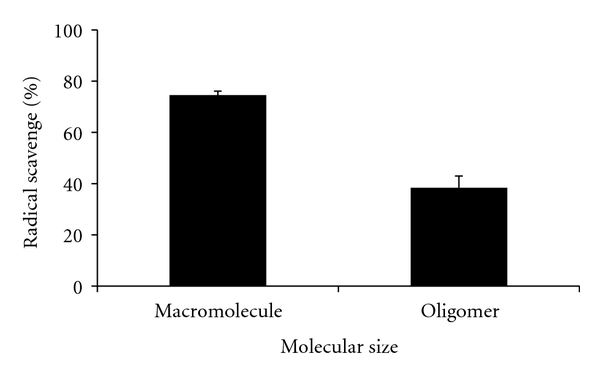
Effect of barley *β*-glucan molecular size on antioxidant activity. *β*-glucan concentration: 1.0 w/v%. Data are presented as mean ± SD (*n* = 3).

The molecular weight of *β*-glucan extracted from barley with warm water is 40,000–100,000 Da; the oligomer prepared from the macromolecule *β*-glucan by enzymatic degradation with lichenase has a molecular weight of approximately 2,000 Da (as described by the manufacturer (ADEKA Co., Tokyo, Japan)). As shown in Figure 5, the hydroxyl radical scavenging activity of *β*-glucan was reduced with a decrease in molecular size. However, antioxidant activity was reduced only by about half, even when molecular weight was reduced by about 1/20–1/50. This finding indicates that *β*-glucan exerts hydroxyl radical scavenging activity across a wide range of molecular sizes. 

### 3.4. Comparison of *β*-Glucan Antioxidant Activity with Other Polymers

**Figure 6:**
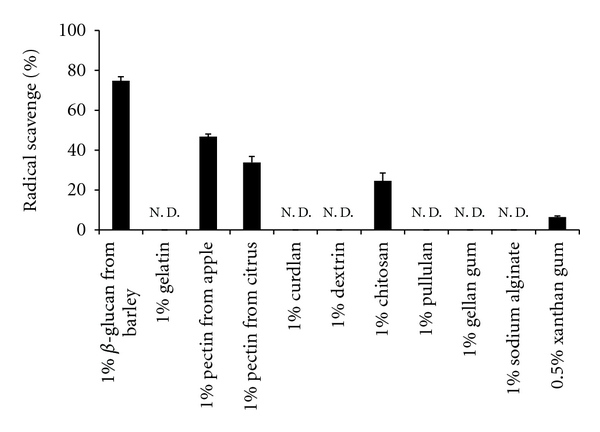
Comparison of barley *β*-glucan antioxidant activity with other polymers. N. D.: not detected. Data are presented as mean ± SD (*n* = 3).

The hydroxyl radical scavenging activity of various polymers was determined and compared with that of *β*-glucan. In this experiment, the hydroxyl radical scavenging activity of 1% xanthan gum could not be determined due to its high viscosity; therefore, 0.5% was used. As shown in Figure 6, pectin from apple or citrus, chitosan, and xanthan gum showed hydroxyl radical scavenging activity. However, the hydroxyl radical scavenging activity of each of these polymers, which are used as food additives, was inferior to that of *β*-glucan extracted from barley. The polymers pullulan, dextrin, and curdlan, which are copolymers of glucose, were also assessed. Pullulan is a linear *α*-1,4 : 1,6-d-glucan consisting predominantly of repeating maltotrioses (consisting of 3 d-glucose molecules linked with *α*-1,4 glycosidic bonds) linked by *α*-1,6-glucosidic bonds. Dextrin is also a *α*-1,4-d-glucan or *α*-1,6-d-glucan, whereas curdlan is a linear *β*-1,3-d-glucan consisting of *β*-(1,3)-linked d-glucose residue. Hydroxyl radical scavenging activity was not observed for curdlan, though curdlan is a *β*-glucan; further, no antioxidative activity was observed for pullulan or dextrin. This finding indicates that the hydroxyl radical scavenging activity of *β*-glucan is affected by the primary structure of the molecule, including linkage type, degree of branching, molecular weight, and conformation.

## 4. Conclusion

Hydroxyl radicals have the strongest reactivity and oxidation power among ROS. The ability to scavenge ROS is a precious property for the prevention of various diseases and aging. In this study, it was shown that *β*-glucan extracted from barley exerts significant antioxidant activity, in addition to the various biologic activities previously described. The amount of antioxidant activity of *β*-glucan was influenced by the different physiologic properties (e.g., structure and molecular size) of *β*-glucan, which varied depending on the source and extraction method used. Furthermore, the hydroxyl scavenging activity of *β*-glucan was significantly higher than that of various polymers that are used as food additives. These results indicate that *β*-glucan has promise as a polymeric excipient for supplement and food additive with antioxidant and other benefits, which may contribute to enhancing health and beauty.
